# Development and validation of a novel immunotype for prediction of overall survival in patients with clear cell renal cell carcinoma

**DOI:** 10.3389/fonc.2022.924072

**Published:** 2022-09-28

**Authors:** Deshui Yu, Xuanzhi Zhang, Lixia Gao, Subo Qian, Hong Tang, Ning Shao

**Affiliations:** ^1^ Department of Urology, Second People’s Hospital of Wuxi Affiliated to Nanjing Medical University, Wuxi, China; ^2^ Department of Urology, Fudan University Shanghai Cancer Center, Shanghai, China; ^3^ Department of Operation Room, Second People’s Hospital of Wuxi Affiliated to Nanjing Medical University, Wuxi, China; ^4^ Department of Urology, Xinhua Hospital, School of Medicine, Shanghai Jiao Tong University, Shanghai, China; ^5^ Department of Pathology, The Affiliated WuXi No.2 People’s Hospital of Nanjing Medical University, Wuxi, China

**Keywords:** ccRCC, immunotype, PD-1, PD-L1, overall survival

## Abstract

**Background:**

Clear cell renal cell carcinoma (ccRCC) is a highly immunogenic tumor. The purpose of the present study was to establish a novel immunotype for different immune infiltration and overall survival (OS) of patients with ccRCC.

**Methods:**

Based on the Cancer Genome Atlas Project (TCGA) database (discovery set), a novel immunotype was established using ssGSEA methods. The databases of Fudan University Shanghai Cancer Center (FUSCC) and Xinhua Hospital Affiliated to Shanghai Jiaotong University School of Medicine (XHH) served as an external validation set. GSEA was carried out to identify the immunotype associated signal transduction pathways.

**Results:**

A total of 652 ccRCC patients were included in our study. We constructed a novel immunotype of ccRCC to classify patients into three groups: high-immunity, moderate-immunity, and low-immunity. The high-immunity and moderate-immunity groups had higher ImmuneScores, ESTIMATEScores, StromalScores, and lower tumor purity than that of the low-immunity group in both sets. Additionally, the patients from the high-immunity and moderate-immunity groups had longer survival than patients from low-immunity group in both discovery set and validation set (HR = 2.54, 95% CI: 1.56–4.13, p < 0.01; HR = 2.75, 95% CI: 1.24–6.11, p = 0.01).

**Conclusion:**

In summary, we defined a novel immunotype of ccRCC. The immune types could be used as a clinical predictive tool to identify ccRCC patients with different survival. In addition, the immune-related biological signaling pathway also brought new insights on the mechanism of ccRCC.

## Introduction

Clear cell renal cell carcinoma (ccRCC) is the most common kidney cancer. Epidemiological studies showed that most ccRCC patients have no metastasis at the initial diagnosis. However, one in five patients may develop metastatic RCC (mRCC) after nephrectomy in 3 years (1–5). Therefore, having signatures that can identify low-risk patients from high-risk ones would have a significant clinical impact. However, previous studies focused on clinical characteristics or gene expression data rather than the immune microenvironment.

ccRCC is a highly immunogenic and vascularized tumor (6). Agents that target angiogenic pathways were the major current therapeutic regimens for advanced ccRCC. These agents include vascular endothelial growth factor receptor (VEGFR) and tyrosine kinase inhibitors (TKIs; sunitinib, cabozantinib, axitinib, pazopanib) (7–9). Furthermore, recent evidence has shown that blocking immune checkpoints, such as inhibition of programmed cell death-1/programmed cell death ligand 1 (PD-1/D-L1), are effective in ccRCC (10–14). In addition, immune checkpoint inhibitors (ICIs) were effective in tumors refractory to chemotherapy such as lung cancer and metastatic melanoma. These new treatments will involve greater personalization to get more benefits for individual patients (15–17). Hence, the immunotype is a potential biomarker and can be applied in ccRCC.

The present study was designed to develop a novel immunotype based on our microarray database and public database. This new immunotype could indicate different immunocyte infiltration of ccRCC patients and predict the prognosis of ccRCC patients. In addition, analysis of the pathway and function of different immunotypes can bring new insights into the underlying molecular mechanism of ccRCC.

## Materials and methods

### Patients’ samples and follow-up

The study was approved by the Ethics Committee of Xinhua Hospital Affiliated to Shanghai Jiaotong University School of Medicine (Shanghai, China) and Fudan University Shanghai Cancer Center (FUSCC). Tissue samples were obtained from patients with ccRCC who underwent operation from February 2011 to June 2016 at Xinhua Hospital and FUSCC. All patients were informed of the importance of follow-up and were regularly followed. The study was performed following the Declaration of Helsinki.

### RNA extraction and microarray assay

Total RNA was extracted from all patients’ tissues from Xinhua Hospital using TRIzol reagent following the manufacturer’s protocol. We measured RNA quantity and quality using a NanoDrop ND-1000 spectrophotometer. The details of the microarray assay are described in our previous study.

### Preparation of datasets

We enrolled 652 patients in our study, including 526 patients from the discovery set (The Cancer Genome Atlas Project (TCGA) database) and 126 patients from the validation set (Xinhua Hospital and FUSCC). In the validation set, 30 patients were from Xinhua Hospital and 96 patients were from FUSCC.

### Construction of the immunotype of ccRCC

A total of 526 ccRCC patients with sufficient clinical information were included in the discovery set. Sixteen immune-associated gene sets which represented diverse immune cell types, functions, and pathways were analyzed. We used the single-sample gene set enrichment analysis GSEA (ssGSEA) to quantify the activity or enrichment levels of immune cells, functions, or pathways in our samples. ccRCC patients in the discovery set were then clustered into three immune types: high-immunity group, moderate-immunity group, and low-immunity group based on Immunescore, ESTIMATEscore, StromalScore. To assess associations between the immunotype and overall survival (OS), Kaplan–Meier curves and log-rank tests were used. To validate the prognostic value of the immunotype, the same analyses were used in our validation set.

### Identification of immunotype associated with signal transduction pathways

To identify the immunotype associated with biological pathways, GSEA was performed, including Gene Ontology (GO) and Kyoto Encyclopedia of Genes and Genomes (KEGG) pathway enrichment analyses. Hence, the immunity-associated biological function or regulation in HCC could be obtained. The FDR < 0.05 and P-value < 0.001 were set as the cutoff criteria.

### Statistical analysis

The relationship between different immune types and clinical features was assessed by the Wilcoxon rank-sum test for continuous variables and the χ (2) test for categorical variables. To assess the associations between different immunotypes and overall survival, Kaplan–Meier curves and log-rank tests were used. Cox proportional hazards analysis was used to assess the relative impacts of different groups. All statistical analyses were performed using R (version 3.4.4, www.r-project.org). All statistical tests were two-sided, and P-value <0.05 was considered statistically significant.

## Results

### Baseline characteristics

A total of 652 patients (526 patients in the discovery set and 126 patients in the validation set) were enrolled in our study. The average age was 60.42e and 56.60 years in the discovery set and validation set, respectively. The baseline characteristics and pathological parameters are summarized in **Table S1**.

### Construction of a novel immunotype of ccRCC

**Figure 1:**
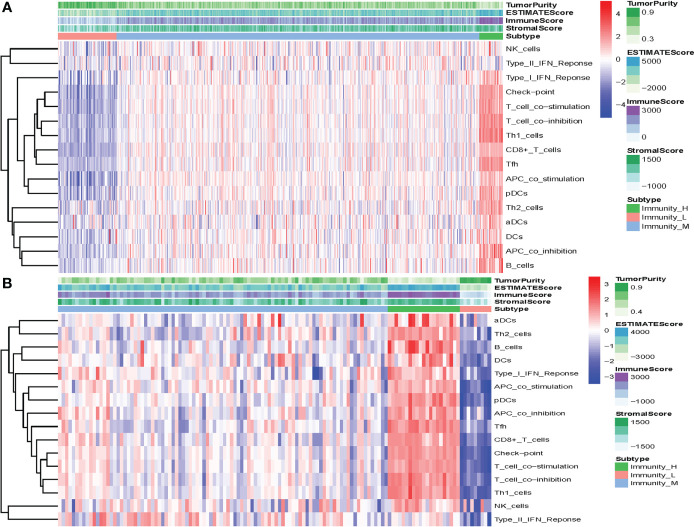
Construction of the immunotype of ccRCC. **(A)** Heat map showed that the patients were clustered into three groups: high-immunity, moderate-immunity, and low-immunity in the discovery set. **(B)** The patients were clustered into high-immunity, moderate-immunity, and low-immunity groups in the validation set using the same method.

**Figure 2:**
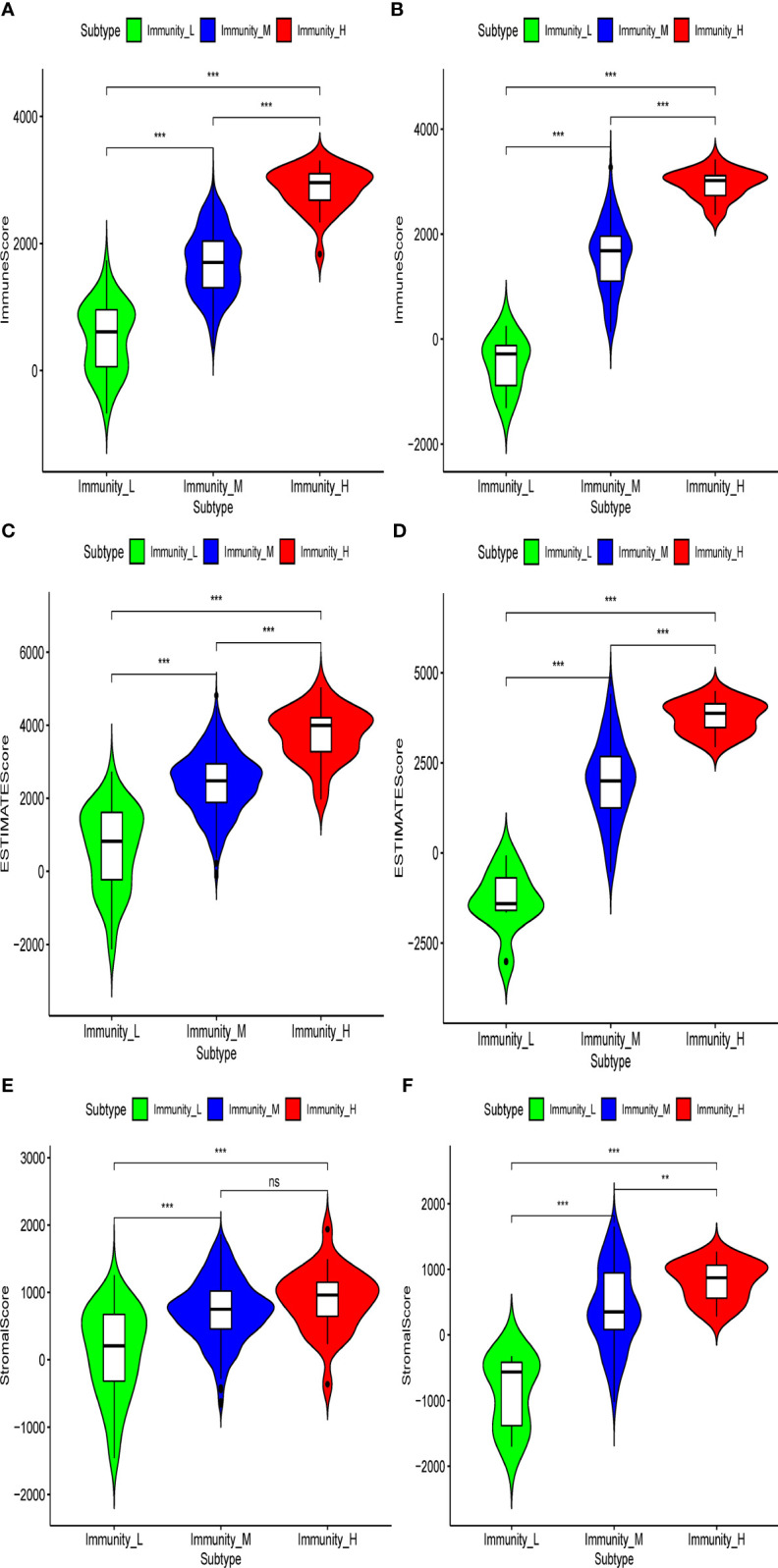
Violin plot showed ImmuneScores, ESTIMATEScores, and StromalScores in different immunity groups in the discovery set and validation set. ImmuneScores in the discovery set **(A)** and validation set **(B)**. ESTIMATEScores in the discovery set **(C)** and validation set **(D)**. StromalScores in the discovery set **(E)** and validation set **(F)**. **p < 0.01; ***p < 0.001. ns, not significant.

**Figure 3:**
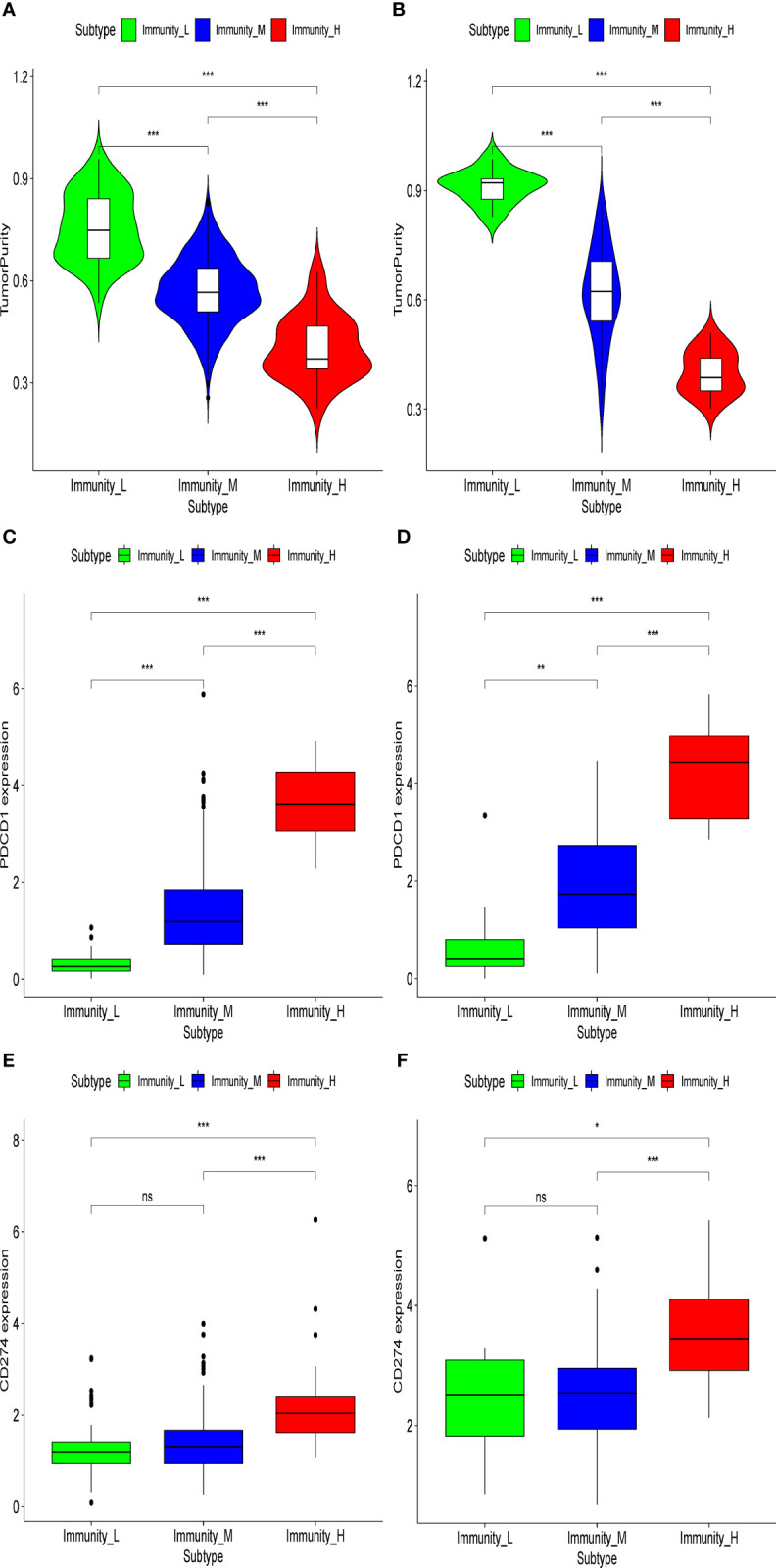
Tumor purity and expression of PD1/PD-L1 in different immunity groups in the discovery set and validation set. Tumor purity in the discovery set **(A)** and validation set **(B)**. Expression of PD1 in the discovery set **(C)** and validation set **(D)**. Expression of PD-L1 in the discovery set **(E)** and validation set **(F)**. *p < 0.05; **p < 0.01; ***p < 0.001. ns, not significant.

In the present analysis, the patients were clustered into three groups (high-immunity, moderate-immunity, and low-immunity) using immune-associated gene sets in the discovery set (**Figure 1A** and **Figure S1A**). There were 69, 429, and 28 patients in the high-immunity, moderate-immunity, and low-immunity groups, respectively. The patients in the high-immunity group and moderate-immunity group had higher ImmuneScores, ESTIMATEScores, and StromalScores (**Figures 2A, C, E**), and lower tumor purity (**Figure 3A**) than in the low-immunity groups (p < 0.05). Interestingly, the expression of PD-L1 and PD-1 of patients from the high-immunity group was significantly higher than that of patients from the moderate-immunity group in the discovery set (**Figures 3C, E**). However, only the expression of PD1 of patients from the moderate-immunity group was significantly higher than that of patients from the low-immunity group. The expression of PD-L1 between the moderate-immunity group and the low-immunity group had no statistical difference (**Figures 3C, E**).

**Figure 4:**
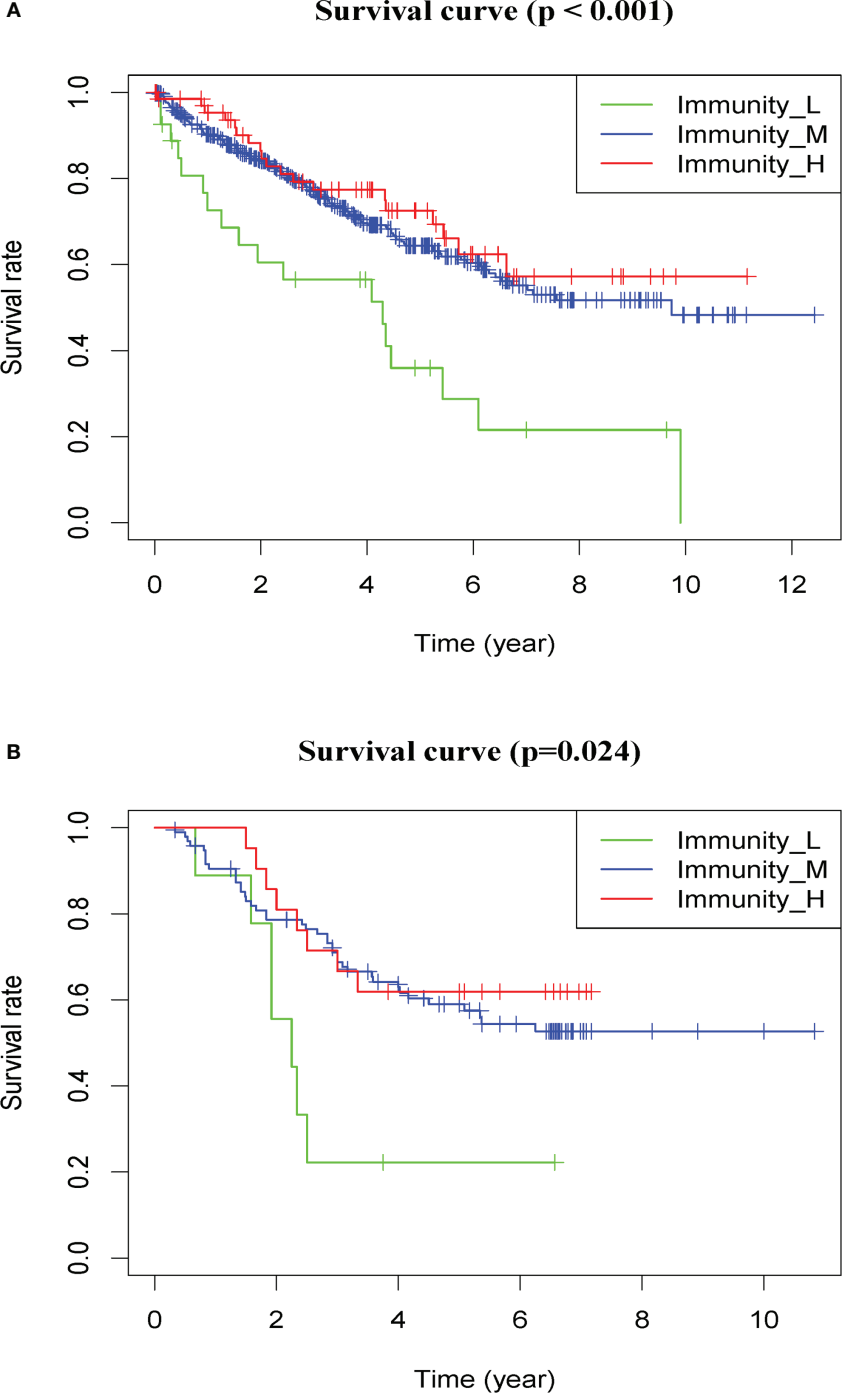
Prognostic value of the immunotype in patients with ccRCC in the discovery set and validation set. Kaplan–Meier survival analysis of overall survival in patients in different groups in the discovery set **(A)** and validation set **(B)**.

In addition, the patients from the high-immunity and moderate-immunity groups had longer survival than patients from the low-immunity group (HR = 2.54, 95% CI: 1.56–4.13, p < 0.01, **Figures 4A** and **S2A**).

### Validation of the prognostic value of the immunotype

In the validation set, there were 21, 96, and nine patients in the high-immunity, moderate-immunity, and low-immunity groups, respectively (**Figures 1B** and **S1B**). Similarly, the ESTIMATEScores, ImmuneScores, and StromalScores of patients in the high-immunity group and moderate-immunity groups were significantly higher than those of patients in the low-immunity group (**Figures 2B, D, F**, p < 0.05). The tumor purity of patients in the high-immunity group and moderate-immunity groups was lower than that of patients in the low-immunity group significantly (**Figure 3B**, p < 0.05). In addition, the expression of PD-L1 and PD-1 of patients in the high-immunity group was higher than that of patients in the moderate-immunity group in our validation set significantly (**Figures 3D, F**). Furthermore, the expression of PD1 between the moderate-immunity group and the low-immunity group had statistical differences, while the expression of PD-L1 between the two groups had no such significant results (**Figures 3D, F**). These results are consistent with those in the discovery set.

The patients from the high-immunity and moderate-immunity groups also had better survival than patients from the low-immunity group (HR = 2.75, 95% CI: 1.24–6.11, p = 0.01, **Figures 4B** and **S2B**).

### Identification of immunotype associated with signal transduction pathways

To investigate the immunity-associated biological signaling pathways in ccRCC, we performed bioinformatics analysis, such as GO and KEGG analyses, in both sets. Significant gene sets (p < 0.05) were shown as Enrichment Map (**Figure S2**)).

**Figure 5:**
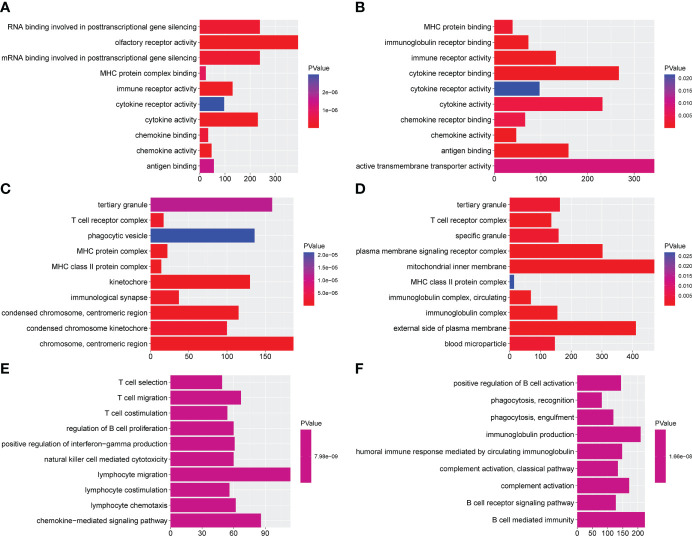
Gene Ontology analysis in the discovery set and validation set. The histogram map of the immunotype-associated molecular function in the discovery set **(A)** and validation set **(B)**, cellular component in the discovery set **(C)**, and validation set **(D)** and biological process in the discovery set **(E)** and validation set **(F)**.

**Figure 6:**
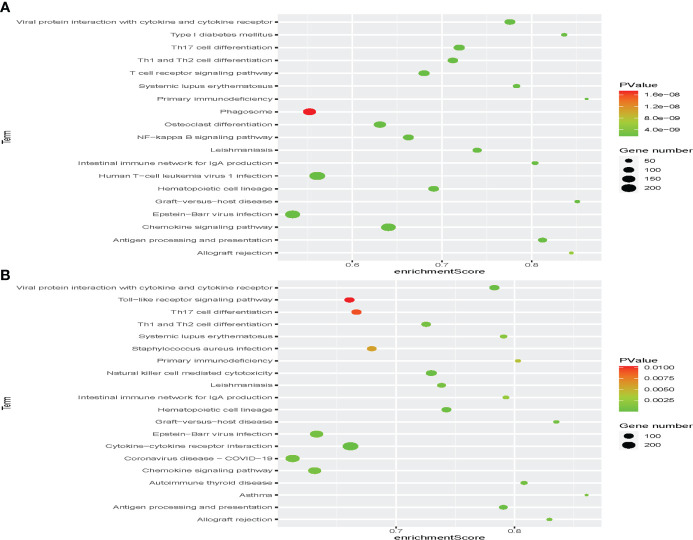
Identification of the immunotype-associated biological signaling pathway. Gene set enrichment analysis (GSEA) in the discovery set **(A)** and validation set **(B)** to identify immunotype-associated signal transduction pathways.

In the discovery set, a total of 792 enriched GO terms were identified, among which 42 belong to molecular function, 58 belong to cellular component, and 692 belong to biological process (**Figure S3A**). The three most enriched molecular function terms were immune receptor activity, cytokine activity, and olfactory receptor activity (**Figure 5A**). When it came to cellular component and biological process, MHC class II protein complex, MHC protein complex, kinetochore and lymphocyte chemotaxis, T-cell selection, and T-cell migration ranked the three most enriched GO terms, respectively (**Figures 5C, E**). In addition, we identified 69 immunity-associated KEGG pathways. Primary immunodeficiency, graft-versus-host disease, type I diabetes mellitus, antigen processing and presentation, and intestinal immune network for IgA production ranked the five most enriched pathways (**Figures 6A** and **S3B**).

In the validation set, a total of 235 enriched GO terms were identified, among which 11 belong to molecular function, 13 belong to cellular component, and 211 belong to biological process (**Figure S3C**). The three most enriched molecular function terms were immunoglobulin receptor binding, antigen binding, and immune receptor activity (**Figure 5B**). When it came to cellular component and biological process, immunoglobulin complex, circulating, immunoglobulin complex, T-cell receptor complex and phagocytosis, recognition, complement activation, and humoral immune response mediated by circulating immunoglobulin ranked the three most enriched GO terms, respectively (**Figures 5D, F**). In addition, we identified 41 immunity-associated KEGG pathways. Asthma, graft-versus-host disease, allograft rejection, autoimmune thyroid disease, and primary immunodeficiency ranked the five most enriched pathways (**Figures 6B** and **S3D**).

Through the comparison, we could find 167 duplicated enriched GO terms, among which seven belong to molecular function, six belong to cellular component, and 154 belong to biological process. The three most enriched molecular function terms were antigen binding, immune receptor activity, and chemokine activity. When it came to cellular component and biological process, plasma membrane signaling receptor complex, T-cell receptor complex, specific granule and antigen receptor-mediated signaling pathway, B-cell-mediated immunity, and B-cell receptor signaling pathway ranked the three most enriched GO terms, respectively. We also identified 37 duplicated immunity-associated KEGG pathways. Viral protein interaction with cytokine and cytokine receptor, natural killer cell-mediated cytotoxicity, antigen processing and presentation, hematopoietic cell lineage, and autoimmune thyroid disease ranked the five most enriched pathways.

## Discussion

ccRCC is the most common renal cancer with a continually increasing incidence and mortality in China. Surgery is the main treatment option for patients with non-metastatic ccRCC. TKIs and ICIs are the major first-line treatment for patients with mRCC (18–20). As 20% patients with non-metastatic ccRCC may develop mRCC after surgery in 3 years, it is critical to develop an effective classification to identify patients at lower risk from those at higher risk. Previous studies focused on the TNM staging system or gene signatures. Recently, immune checkpoint inhibitors have been at the forefront position in cancer research. A few studies suggested that immune landscape was associated with the prognosis of patients with several types of solid tumors, such as breast cancer and lung cancer (21–23). Thus, the latest articles also investigated the role of the tumor immune microenvironment in ccRCC progression and metastasis (10, 24). Komohara and Chevrier found that many immune cells, such as NK cells and T cells (including CD8+ T cells, CD4+ T cells), were associated with prognosis of ccRCC (25–27).

Additionally, the prognosis of PD1/PD-L1 in various cancers has been a research hotspot. Iacovelli indicated that a high expression of PD-L1 was a negative prognostic factor in RCC (28). Shen suggested that PD-L1 is a novel prognostic marker for RCC and is associated with poor prognosis (29). Based on these latest research, a few studies have developed various risk stratification models to classify RCC patients into different groups with different prognoses in recent years. Feng constructed an immune prognostic signature that included 14 immune-associated genes in 2021 (30). Their result suggested that their immune signature was an independent prognostic factor for ccRCC. This immune risk score was also correlated with significant immunophenotypic factors, such as T-cell infiltration and antitumor response. Bassanelli also built a 17-gene expression signature of patients with ccRCC in 2021 (5). This signature was correlated with recurrence-free survival and overall survival of patients with ccRCC. There are many similar signatures with a common characteristic. The establishment of the risk score was calculated using some coefficients to multiply the expression levels of selected genes. This algorithm was too literally or mechanically with low reproducibility. Therefore, our study used ssGSEA methods based on 16 immune-associated gene sets to construct a novel immunotype of ccRCC instead of calculating risk scores mechanically.

The results indicated that our novel immunotype could identify different immunocyte infiltrations of ccRCC patients in both discovery set and validation set. Patients could be clustered into high-immunity group, moderate-immunity group, and low-immunity group. Patients with various infiltrations of immunocytes had different prognoses. In both sets, patients in the high-immunity and moderate-immunity groups had better OS than those in the low-immunity group significantly. The immunotype may represent a valuable biomarker for ccRCC.

It is widely known that the clinical and pathological stages were significantly associated with prognosis of ccRCC. However, assigning the clinical stage, especially for the pathological stage, had many confounding factors such as subjectivity, sampling error, and interobserver variability. Our immunotype was constructed by gene expression using sequencing technology and ssGSEA methods. The assessment of our immunotype was more objective and could not combined with the clinical stage.

Currently, several phase III trials show the superiority of combination therapy, dual immunotherapy (ICI-ICI), or ICI plus tyrosine kinase inhibitors (TKI) of the vascular endothelium growth factor receptor (VEGFR) over sunitinib monotherapy. The results led to the approval of ICI-ICI and ICI-TKI combinations in first-line treatment (31–33). In addition, immunotherapy has recently also been available in the adjuvant setting based on the results of the first positive phase 3 study of adjuvant immunotherapy for patients with RCC at intermediate-high or high risk of recurrence after nephrectomy or nephrectomy and resection of metastatic lesions (33). These trials suggested that the patients who responded to immunotherapy may have a long-term and durable benefit.

Therefore, additional biomarkers could help in predicting outcomes and benefits of ICI monotherapy or ICI–ICI combinations. Previous studies found that PD-L1 may be a biomarker of PFS but not OS for patients who received ICI monotherapy or TKI–ICI combinations. Besides, many issues like the definition of PD-L1 positivity and using different antibodies may confound PD-L1 biomarker data. Hence, PD-L1 was not an ideal biomarker and a composite biomarker may be required. Our novel immunotype brought new inspiration into this area. Nevertheless, our study also had some limitations. First, the sample size in our validation set was not as large as that of the discovery set. Additionally, the lack of complete data in ICI monotherapy or ICI–ICI combinations trials for testing our novel immunotype was a regrettable thing. This work may be done well in future clinical trials.

## Conclusion

In summary, we defined a novel immunotype of ccRCC using ssGSEA based on immune cell infiltration. Additionally, the patients from the high-immunity group and moderate-immunity group had a better prognosis than patients from the low-immunity group. The immune types could be used as a clinical predictive tool to identify ccRCC patients with different survival. It might also help in the decision-making of personalized therapy and individualized follow-up after surgery. In addition, the immune-related biological signaling pathway also gave new insights on the mechanism of ccRCC.

## Data availability statement

The datasets presented in this study can be found in online repositories. The names of the repository/repositories and accession number(s) can be found in the article/**Supplementary Material**.

## Ethics statement

The study was approved by the Ethics Committee of Xinhua Hospital Affiliated to Shanghai Jiaotong University School of Medicine (Shanghai, China) and Fudan University Shanghai Cancer Center (FUSCC). The patients/participants provided their written informed consent to participate in this study.

## Author contributions

NS had full access to all of the data in the study and take responsibility for the integrity of the data and the accuracy of the data analysis. Concept and design: HT and NS. Acquisition, analysis, or interpretation of data: DY and XZ. Drafting of the manuscript: LG and SQ. Critical revision of the manuscript for important intellectual content: HT and NS. All authors contributed to the article and approved the submitted version.

## Conflict of interest

The authors declare that the research was conducted in the absence of any commercial or financial relationships that could be construed as a potential conflict of interest.

## Publisher’s note

All claims expressed in this article are solely those of the authors and do not necessarily represent those of their affiliated organizations, or those of the publisher, the editors and the reviewers. Any product that may be evaluated in this article, or claim that may be made by its manufacturer, is not guaranteed or endorsed by the publisher.
