# Supplementary material for: Development and validation of a novel immunotype for prediction of overall survival in patients with clear cell renal cell carcinoma
Source: Front Oncol. 2022 Sep 28;12:924072. doi: 10.3389/fonc.2022.924072 (PMC9552763; doi:10.3389/fonc.2022.924072)
Supplement: Supplementary file 1 [file DataSheet_1.pdf]

Supplementary Figure

Figure S1. Construction of the immunotype of ccRCC.

(A) Cluster diagram showed that the patients were clustered into three groups: high-immunity, moderate-immunity and low-immunity in discovery set. (B) The patients were clustered into high-immunity, moderate-immunity and low-immunity group in validation set using the same methed.

Figure S2. Prognostic value of the immunotype in patients between high-immunity +moderate-immunity and low-immunity in discovery set and validation set.

Kaplan–Meier survival analysis of overall survival in patients between high-immunity+moderate-immunity and low-immunity in discovery set (A) and validation set (B).

Figure S3. Identification of the immunotype associated with signal transduction pathways.

(A) The Enrichment Map of the immunotype associated biological signaling pathway in discovery set.

(B) Kyoto Encyclopedia of Genes and Genomes (KEGG) pathway analyses showed that notable pathway of the immunotype in discovery set.

(C) The Enrichment Map of the immunotype associated biological signaling pathway in validation set.

(D) Kyoto Encyclopedia of Genes and Genomes (KEGG) pathway analyses showed that notable pathway of the immunotype in validation set.

Figure S1

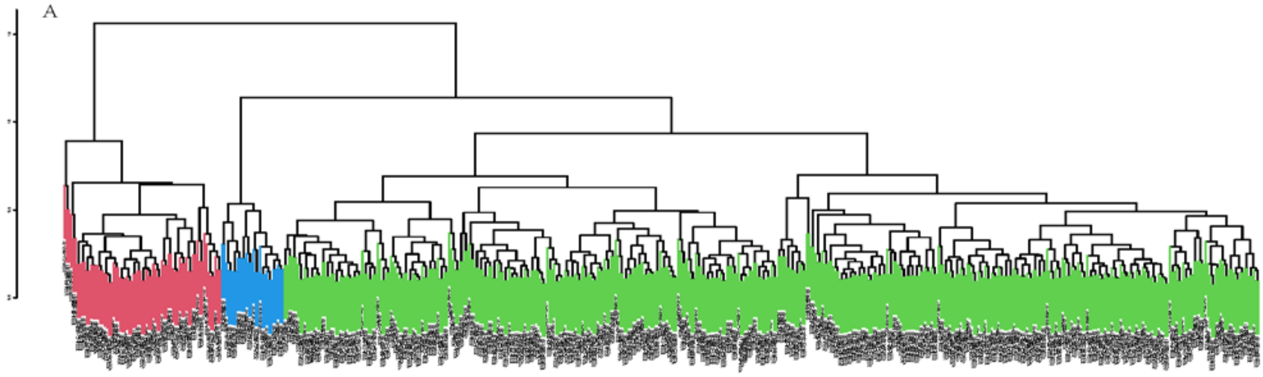

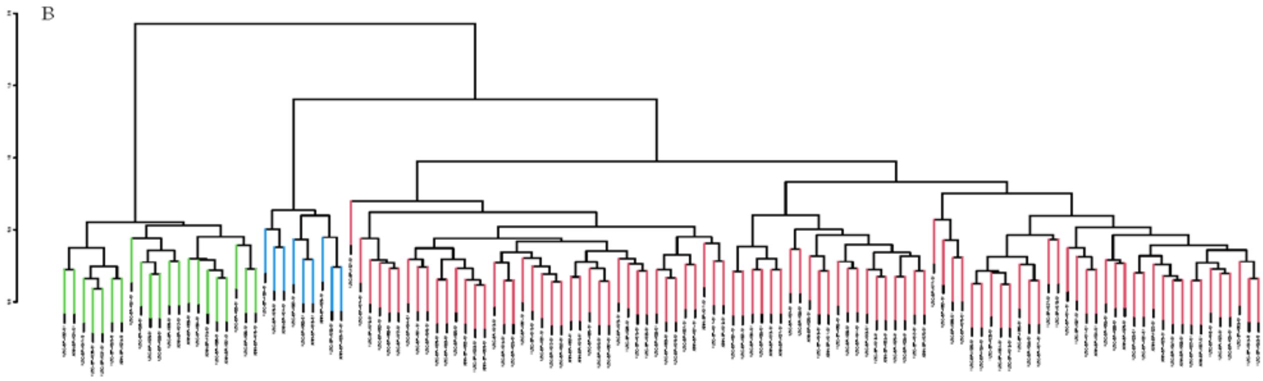

Figure S2

A

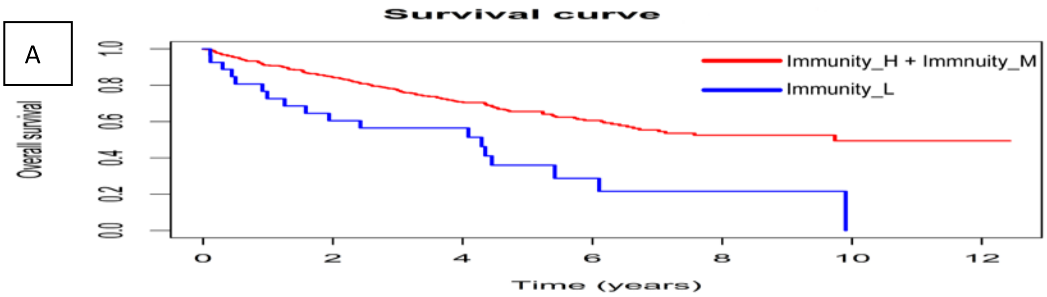

B

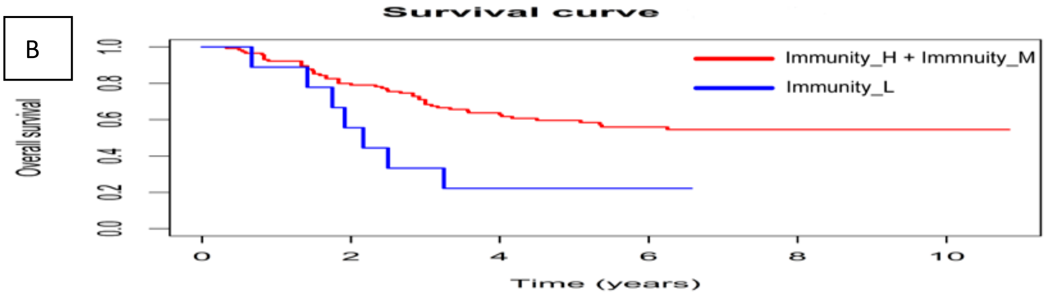

Figure S3

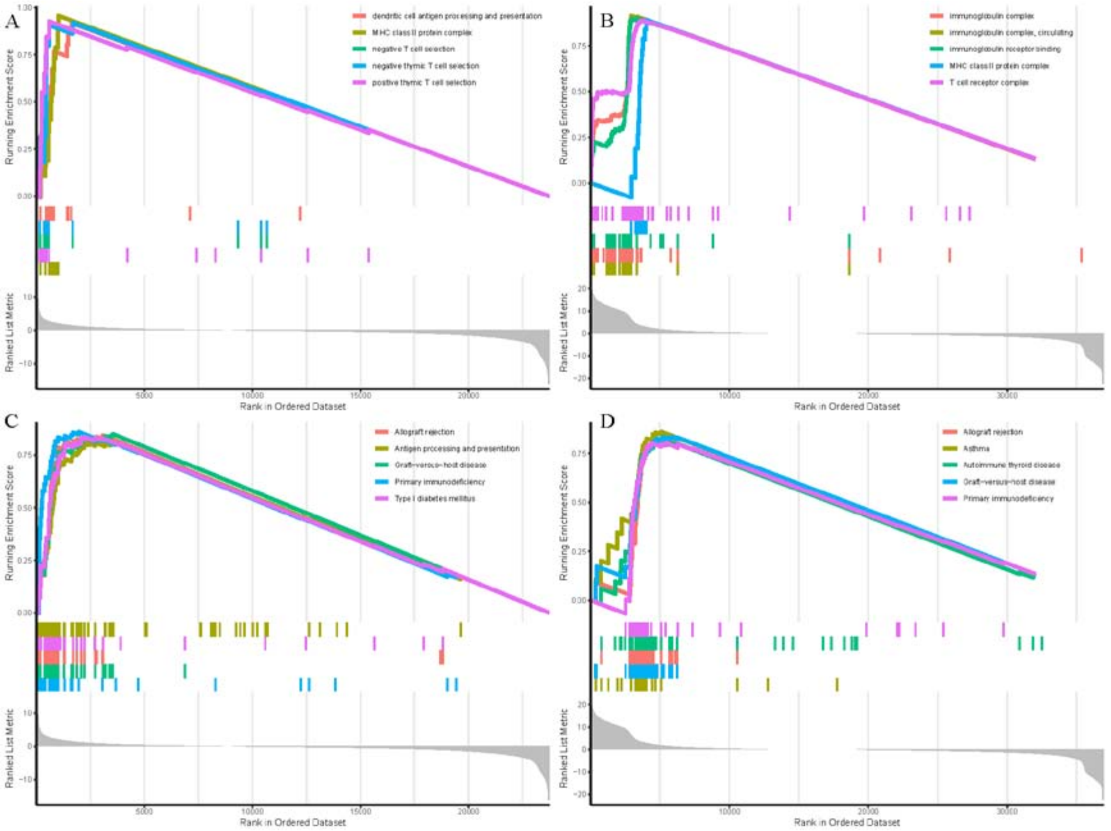
A
dendritic cell antigen processing and presentation
MHC class II protein complex
negative T cell selection
negative thymic T cell selection
positive thymic T cell selection
Running Enrichment Score
Ranked List Metric
Rank in Ordered Dataset
B
immunoglobulin complex
immunoglobulin complex, circulating
immunoglobulin receptor binding
MHC class II protein complex
T cell receptor complex
C
Allograft rejection
Antigen processing and presentation
Graft-versus-host disease
Primary immunodeficiency
Type I diabetes mellitus
D
Allograft rejection
Asthma
Autoimmune thyroid disease
Graft-versus-host disease
Primary immunodeficiency
