# Supplementary material for: Development and validation of a novel immunotype for prediction of overall survival in patients with clear cell renal cell carcinoma
Source: Front Oncol. 2022 Sep 28;12:924072. doi: 10.3389/fonc.2022.924072 (PMC9552763; doi:10.3389/fonc.2022.924072)
Supplement: Supplementary file 2 [file Table_1.docx]

Supplementary Table 1

The baseline characteristics and pathological parameters in discovery set and validation set.

|  | Discovery Set | (N=526) |  | Validation set | (N=126) |
| --- | --- | --- | --- | --- | --- |
|  | TCGA cohort | |  | FUSCC+Xinhua cohort | |
| Variables |  | |  |  | |
| Age |  |  |  |  |  |
| <60 | 245 |  |  | 72 |  |
| ≥60 | 281 |  |  | 54 |  |
|  |  |  |  |  |  |
| Gender |  |  |  |  |  |
| male | 343 |  |  | 79 |  |
| female | 183 |  |  | 47 |  |
|  |  |  |  |  |  |
| Stage |  |  |  |  |  |
| I | 261 |  |  | 62 |  |
| II | 57 |  |  | 21 |  |
| III | 122 |  |  | 11 |  |
| IV | 82 |  |  | 32 |  |
| NA | 3 |  |  |  |  |

FUSCC: Fudan University Shanghai Cancer Center

Xinhua: Xinhua Hospital Affiliated to Shanghai Jiaotong University School of Medicine
